# Evaluation and Treatment of Depression in Stroke Patients: A Systematic Review

**DOI:** 10.7759/cureus.28137

**Published:** 2022-08-18

**Authors:** Vamsi Krishna Lavu, Rana Abdelwahab Mohamed, Ruimin Huang, Shanthi Potla, Sushen Bhalla, Yousif Al Qabandi, Savitri Aninditha Nandula, Chinmayi Sree Boddepalli, Sai Dheeraj Gutlapalli, Lubna Mohammed

**Affiliations:** 1 Internal Medicine, California Institute of Behavioral Neurosciences & Psychology, Fairfield, USA; 2 Dermatology, California Institute of Behavioral Neurosciences & Psychology, Fairfield, USA

**Keywords:** depression, brain stroke, cerebrovascular accidents (cva), post-stroke depression, stroke

## Abstract

Those who received early diagnosis and treatment for poststroke depression had lower mortality rates, cognitive impairments, improved long-term disability, a higher quality of life, and lower rates of suicidal thoughts than those who did not. The Preferred Reporting Items for Systematic Reviews and Meta-Analyses (PRISMA) 2020 standards were used to conduct this systematic review. Until May 1, 2022, a systematic search was conducted utilizing ScienceDirect, Cochrane, PubMed, Google Scholar, and PubMed central databases, which have been used during the previous 10 years. Randomized controlled trials (RCTs), observational studies, systematic reviews, review articles, case reports, clinical studies, and meta-analyses were included in the research, which covered post-stroke depression patients and how to identify and treat them.

There were 545 possibly related titles found in the database search. Finally, each publication was given a quality rating, and 10 studies with a score of higher than 70% were allowed into the review. Because of their brevity and ease of use, they employed the Patient Health Questionnaire-9 (PHQ-9) and PHQ-2 screening instruments in stroke patients. According to pooled studies, the risk of acquiring post-stroke depression (PSD) was lower in participants undergoing pharmacological therapy with selective serotonin reuptake inhibitors (SSRIs), especially after a year. Identifying further features of the PSD process, we believe, is the most pressing need for future study since it might lead to a more precise treatment strategy.

## Introduction and background

Among the leading causes of death worldwide and disability-adjusted life years, stroke ranks second [1]. Following that, in addition to physical disabilities, cognitive and psychological problems may also be present [1]. Stroke rates range from 10 to 20 per 10,000 people in the 50 to 64 age group, to 200 per 10,000 people in the over 85 age group [2]. As well as being a significant public health problem, stroke continues to be a major financial hardship for patients and their families, despite advances in prevention and therapy [1].

The most prevalent psychological condition following a stroke is poststroke depression (PSD) [3]. Poststroke depression affects approximately 85% of strokes and is linked to more severe functional dysfunction, poor rehabilitation outcomes, and social isolation after stroke [4]. Depressed mood apathy, weight loss or increase, sleep disturbances, exhaustion, worthlessness, and anhedonia are the primary clinical signs of poststroke depression, with the first two being the most prominent [5]. The pooled frequency of poststroke depression was 31% in a meta-analysis of 61 studies with 25,488 stroke patients, although it dropped to 25% one to five years after the stroke [6].

Stroke survivors with poststroke depression had a higher chance of poor functional recovery, recurrent episodes of cerebrovascular events, diminishing quality of life, and death than stroke survivors without depression [3]. When compared to post-stroke patients without depression, people with poststroke depression death rates are higher, cognitive deficits are more evident, long-term disability is more prevalent, quality of life is lower, and suicidal ideation is more prevalent, implying that early detection and treatment of depression are critical after a stroke [1]. Patient's quality of life after a stroke is greatly reduced when they have depression and a stroke together [7]. Mood depression, in particular, is thought to be the main determinant of quality of life in stroke survivors [8]. However, anxiety, irritability, agitation, emotional incontinence, modification of the emotional experience, sleep disturbances, behavioral disturbances such as disinhibition, apathy, fatigue, and psychotic symptoms such as delusions and hallucinations are just some of the neuropsychiatric symptoms that can appear after a stroke [9]. Some studies imply that stroke and depression have a bidirectional link. Although stroke increases the risk of depression following a stroke, depression is an independent risk factor for stroke and stroke-related death as well [1].

The Center for Epidemiological Studies Depression Scale (CESD) and the Hamilton Depression Rating Scale have both been used to assess the severity of poststroke depression [6]. Poststroke depression is diagnosed using five criteria: (a) symptoms are pathophysiologically connected to the stroke; (b) symptoms are not better explained by other psychiatric diseases; (d) disruption does not occur just in the context of delirium; (e) symptoms produce severe suffering or impairment [1]. A number of observational studies have indicated that poststroke depression could increase the risk of stroke outcomes [10]. A network approach to studying depression allows researchers to look at possible causal routes among distinct symptoms, as well as how these symptoms may reinforce one another and build feedback loops (e.g., insomnia → fatigue→ not feeling good → insomnia) [6].

This systematic review will look at how to recognize poststroke depression, as well as its prevalence, and links to physical, cognitive, and mortality impairments. We will also talk about how to treat poststroke depression, how to avoid it, what causes it, and what research we should do next.

## Review

Methods

This systematic review followed the Preferred Reporting Items for Systematic reviews and Meta-Analyses (PRISMA) 2020 guidelines [11].

Search Strategy

The PubMed, PubMed Central (PMC), Cochrane, Google Scholar, and ScienceDirect databases were all used to search. The search for all databases was completed on May 1, 2022. As shown in Table 1, the field search employed in the procedure was chosen based on keywords used in prior publications and Medical Subject Headings (MeSH), depending on the database used.

**Table 1:** The approach for doing a bibliographic search in databases, as well as the filters that go with it. PMC - PubMed Central

Databases	Keywords	Search Strategy	Filters	Search Results
PubMed	Stoke, Cerebrovascular accident, cerebral infarction, Depression, Sadness, Unhappiness	#1 Stoke OR Cerebrovascular accident OR cerebral infarction OR ( "Stroke/diagnosis"[Majr] OR "Stroke/metabolism"[Majr] OR "Stroke/pathology"[Majr] OR "Stroke/physiology"[Majr] OR "Stroke/physiopathology"[Majr] OR "Stroke/prevention and control"[Majr] OR "Stroke/psychology"[Majr] OR "Stroke/rehabilitation"[Majr] ) #2 Depression OR Sadness OR Unhappiness ( "Depression/analysis"[Majr] OR "Depression/classification"[Majr] OR "Depression/complications"[Majr] OR "Depression/diagnosis"[Majr] OR "Depression/diagnostic imaging"[Majr] OR "Depression/economics"[Majr] OR "Depression/epidemiology"[Majr] OR "Depression/etiology"[Majr] OR "Depression/genetics"[Majr] OR "Depression/history"[Majr] OR "Depression/pathology"[Majr] OR "Depression/physiology"[Majr] OR "Depression/physiopathology"[Majr] OR "Depression/prevention and control"[Majr] OR "Depression/psychology"[Majr] ) #1 AND #2 - 137	Free full text, Meta-analysis, Systematic Review, Review articles, case reports, clinical study, Randomized Controlled Trials, observational studies, last 10 years, English.	135
PMC	Stroke, Post-stroke Depression, Depression, cerebrovascular accidents	Stroke AND Depression AND Cerebrovascular accidents - 1384	Open access, last 5 years	78
Cochrane	Stroke, Post-stroke Depression, Depression, Brain stroke, Depression Treatment	Stroke AND Depression AND Brain stroke - 43	January 1, 2011 – May 1, 2022	13
Science Direct	Stroke, Post-stroke Depression, Depression, Depression Treatment	Post Stroke Depression AND Depression Diagnosis - 7646	2017 - 2022, Review articles, Research articles, Medicine & Dentistry	147
Google Scholar	Stroke, Post-stroke Depression, Depression, Cerebrovascular accidents, Post-stroke Depression Treatment	Post Stroke Depression AND Post-Stroke Depression Treatment - 20600	2017 - 2022, review articles	172

All references were organized and alphabetized in Endnote Citation manager and Microsoft Excel 2021 (Microsoft, Redmond, WA, USA)was used to remove duplicates. The records were initially examined based on titles and abstracts, with irrelevant research being eliminated. The full-text articles were then obtained. The absence of analysis required for this evaluation led to the exclusion of study procedures. The scientists decided not to remove systematic reviews, meta-analyses, and narrative reviews from the analysis because there were so few in the field. We excluded the papers that are not in English.

Risk of Bias in Individual Studies

According to the type of study, all remaining full articles were evaluated for quality and bias using the following tools: Newcastle Ottawa Tool (NOS); Assessment of Multiple Systematic Reviews 2 (AMSTAR 2); and Narrative reviews, Scale for the Assessment of Narrative Review Articles 2 (SANRA 2), Cochrane Collaboration Risk of Bias Tool (CCRBT) [12-15]. Each evaluation instrument has its own set of criteria and scoring system. When a tool gets a "LOW RISK," "YES," or "PARTIAL YES," or "1," it gets a point. When the number "2" is used, two points are awarded. Each assessment instrument required a score of at least 70% to be acceptable (Table 2). The quality of the final papers selected was also verified by a second author to decrease the risk of bias.

**Table 2:** Each sort of study is evaluated for its quality. NOS - Newcastle Ottawa Scale, AMSTAR 2 - Assessment of Multiple Systematic Reviews 2, SANRA 2 - Scale for the Assessment of Narrative Review Articles 2, CCRBT - Cochrane Collaboration Risk of Bias Tool, RCTs - Randomized Controlled Trials, PICO - Patient or Problem, Intervention, Comparision, Outcome, RoB - Risk of Bias

Quality Assessment Tool	Type of Study	Items and their characteristics	Total score	Accepted score (>70%)	Accepted studies
AMSTAR 2 [12]	Systematic Reviews	Sixteen items: did the research questions and inclusion criteria for the review include the components of PICO? Did the report of the review contain an explicit statement that the review methods were established before the conduct of the review and did the report justify any significant deviations from the protocol? Did the review authors explain their selection of the study designs for inclusion in the review? Did the review authors use a comprehensive literature search strategy? Did the review authors perform study selection in duplicate? Did the review authors perform data extraction in duplicate? Did the review authors provide a list of excluded studies and justify the exclusions? Did the review authors describe the included studies in adequate detail? Did the review authors use a satisfactory technique for assessing the risk of bias (RoB) in individual studies that were included in the review? Did the review authors report on the sources of funding for the studies included in the review? If meta-analysis was justified did the review authors use appropriate methods for the statistical combination of results? If a meta-analysis was performed did the review authors assess the potential impact of RoB in individual studies on the results of the meta-analysis or other evidence synthesis? Did the review authors account for RoB in individual studies when interpreting/discussing the results of the review? Did the review authors provide a satisfactory explanation for, and discussion of, any heterogeneity observed in the results of the review? If they performed quantitative synthesis did the review authors carry out an adequate investigation of publication bias (small study bias) and discuss its likely impact on the results of the review? Did the review authors report any potential sources of conflict of interest, including any funding they received for conducting the review? Scored as YES or NO. Partial Yes was considered as a point.	16	12	Wu, Q. E et al. 2019 [3] Zhang, Y et al. 2017 [4]
SANRA 2 [13]	Narrative review	Six items: justification of the article’s importance to the readership, statement of concrete aims or formulation of questions, description of the literature search, referencing, scientific reason, and appropriate presentation of data. Scored as 0,1, or 2.	12	9	Feng, C et al. 2014 [5] Alajbegovic, A et al. 2014 [7] Espárrago Llorca, G et al. 2015 [9]
NOS [14]	Non-randomized Control Trials and Observational studies	Eight items: representativeness of the exposed cohort, selection of the non-exposed cohort, ascertainment of exposure, a demonstration that outcome of interest was not present at the start of the study, comparability of cohorts based on the design or analysis, assessment of outcome, was follow-up long enough for outcomes to occur, adequacy of follow-up of cohorts. Scoring was done by placing a point on each category and scored as 0, 1, or 2. A maximum of two points are allotted in this category.	9	7	López-Espuela, F et al. 2020 [1] Ashaie, S. A et al. 2021 [6] Cai, W et al. 2018 [10]
CCRBT [15]	RCTs	Seven items: random sequence generation and allocation concealment (selection bias), selective outcome reporting (reporting bias), other sources of bias, blinding of participants and personnel (performance bias), blinding of outcome assessment (detection bias), and incomplete outcome data (attrition bias). Bias is assessed as LOW RISK, HIGH RISK, or UNCLEAR.	7	5	Robinson, R. G., & Jorge, R. E. 2016 [2] Paolucci S. 2008 [8]

Results 

Study Selection and Quality Assessment

**Figure 1:**
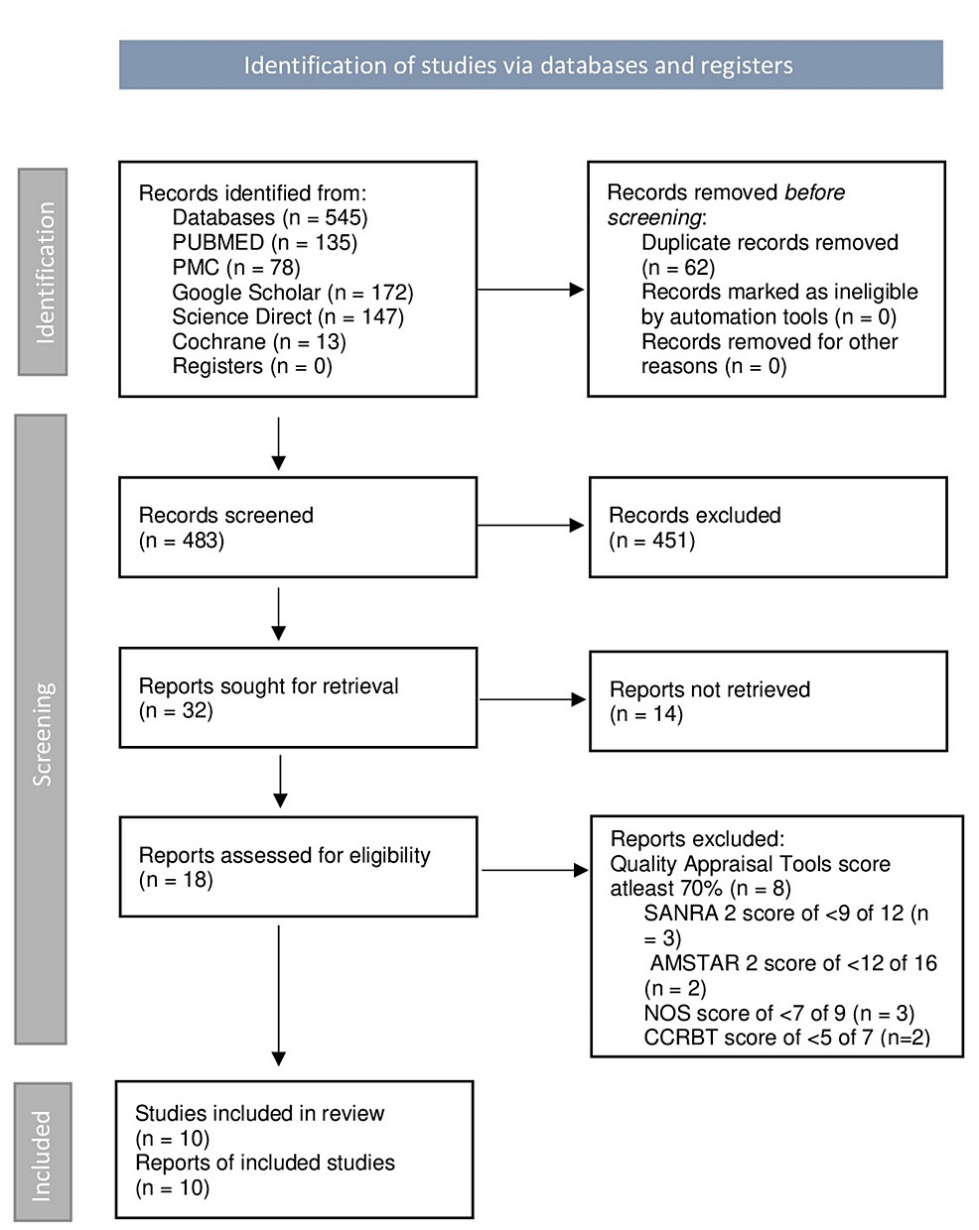
A flowchart depicting the research search selection process. NOS - Newcastle Ottawa Scale, AMSTAR 2 - Assessment of Multiple Systematic Reviews 2, SANRA 2 - Scale for the Assessment of Narrative Review Articles 2, CCRBT - Cochrane Collaboration Risk of Bias Tool, n - number of records, PMC - PubMed Central

There were 545 possibly related titles found in the database search. In Microsoft Excel 2021, duplicates were removed and 483 records were kept. When the titles and abstracts of these records were evaluated using the problem, intervention, and outcome (PIO) components and eligibility criteria of this review, 32 publications remained. These papers were located, and 14 research procedures were found to be ineligible. Finally, each publication was given a quality rating, and the review considered 10 studies with scores over 70% (Figure 1). The quality of the final papers selected was also verified by a second author to decrease the risk of bias. There were no more resources uploaded.

Discussion

Robinson and his colleagues have mentioned the normal path of depression following stroke, with remission seen spontaneously in one to two years after the stroke. However, it was also reported that in a small number of individuals, depression develops chronic and can last for up to three years after a stroke. Minor depression, on the other hand, proved to be more varied, with individuals experiencing both short and long-term despair. Poststroke depression is frequent in both men and women, but when the frequency of the two sexes is compared, it appears that poststroke depression is more common in women [7,16].

Due to its effects on levels of disability and independence, the severity of the stroke was identified as a crucial component of poststroke depression (PSD), which should be prevented by reducing its risk [17]. Patients' self-confidence may be lowered as a result of movement abnormalities, malfunction, and life barriers caused by brain injury, and a sudden stroke may be viewed as a negative experience for sufferers, thereby increasing the prevalence of depression [1].

The magnitude of the infarction is a feature of the lesion that may be linked to the existence and severity of poststroke depression. A bigger infarction size leads to more extensive damage in the brain involving biochemical changes and emotional behavior modulation. A major social-psychological element linked to the development of poststroke depression might be the significant neurological deficiency induced by a massive infarction. The relationship between infarction size and poststroke depression has been studied in several research papers. In a study of 70 individuals with single lesions, Vataja et al. [18] found that lesions in the frontal-subcortical circuits were greater in patients with poststroke depression than in other stroke patients. Nys et al. [19] observed that early poststroke depression was strongly linked with lesion size (P = 0.008) in a prospective analysis of 126 participants. The volume of acute infarcts was larger in the poststroke depression group than in the control group (P = 0.029) in a Chinese cohort study of poststroke depression [20]. Unlike the contentious evidence for the lesion site theory, the findings on the links between poststroke depression and infarction size appeared to be universal in support of the conclusion that large infarction sizes are linked to poststroke depression [5].

Diagnosis

A patient with a significant depression-like episode due to a stroke must have a low mood or a lack of interest or pleasure, as well as four additional signs of depression, for at least two weeks. Patients with a diagnosis of mood disorder due to stroke with depressive aspects must have a sad mood or a lack of interest or pleasure, as well as at least two but not more than five significant depression symptoms lasting two weeks or more [2].

The diagnostic value of the Patient Health Questionnaire (PHQ) in its two-item Patient Health Questionnaire-2 (PHQ-2) and nine-item Patient Health Questionnaire-9 (PHQ-9) variants are investigated and compared to other scales, such as the Hospital Anxiety and Depression Scale (HADS), the Beck Depression Inventory-II (BDI-II), the Distress Thermometer (DT), and the Kessler-10 (K-10), in patients with acute stroke and preserved communication skills, according to a study published in the journal Stroke [21]. The PHQ-9 covers all nine Diagnostic and Statistical Manual of Mental Disorders (DSM) defined symptoms of depression, whereas the PHQ-2 only includes anhedonia and low mood. They advise utilizing PHQ-2 in all patients and PHQ-9 only in those who have PHQ-2 values that are positive since this has the maximum sensitivity. Because of their brevity and ease of use, they recommend these two screening instruments for stroke patients, with the caveat that they cannot be utilized in individuals with considerable cognitive and language impairment (Table 3) [9].

**Table 3:** PHQ-2 is a quality-of-life survey (Patient Health Questionnaire-2). Pathological = > 2

Name	Visit Day			
In the last 2 weeks, how many times have you been concerned about the following problems?	Never	Some Days	More than half the days	Almost every day
Little interest or pleasure in doing things	0	1	2	3
Feeling down, depressed, hopeless	0	1	2	3

Treatment

Pharmacotherapy is currently the most popular form of treatment for poststroke depression, especially during the subacute stage after a stroke. In actuality, it takes several weeks before any effects of development can be shown after a psychotherapy session, in addition to being expensive in terms of employee time and experience. In a rehabilitation program with a set time constraint, this delay might be crucial. As a result, in most clinical settings, the first most practical choice is antidepressant therapy, with psychotherapy intervention being saved for those for whom antidepressants are either inappropriate or useless. Methodological constraints in extant research hinder a conclusion on the effectiveness of psychotherapeutic therapies, as each psychological intervention has empirical support for its usefulness [22,23]. Cognitive-behavioral treatment, on the other hand, has demonstrated some promising effects that merit further investigation [24,25].

Nortriptyline, a tricyclic medication, has been studied in two randomized, double-blind, placebo-controlled investigations [26,27]. The efficacy of fluoxetine was likewise evaluated in the previous one [27]. In all investigations, the treated groups showed a substantial reduction in depressive symptoms when compared to the control groups. In the first research, individuals treated with nortriptyline saw a 79% improvement in Hamilton Depression Rating Scale (HDRS) score from baseline to endpoint, compared to a 40% improvement in those treated with placebo [26,27]. In the second research, depressed individuals receiving nortriptyline saw a greater average improvement in HDRS score (60% vs. 9% for patients receiving fluoxetine and 30% for patients receiving placebo). [27]. Furthermore, the nortriptyline group showed a stronger recovery in daily living activities in Robinson and colleagues' study, while not in Lipsey and Robinson's. However, there was some dispute on the drop-out rate, which was greater in the Lipsey and Robinson research (38%) and lower in the Robinson and colleagues study (40%) among those treated with nortriptyline. However, in this latter trial, the large drop-out rate might have been caused by the fluoxetine dosage, which was used up to 40 mg per day [27]. Furthermore, the findings of this study sparked a discussion concerning methodological issues [28].

The efficacy of citalopram, a selective serotonin reuptake inhibitor (SSRI), was evaluated in two double-blind controlled experiments, the first comparing it to a placebo and the second to the noradrenergic medication reboxetine [29,30]. Citalopram was shown to be effective and tolerable in both investigations. In the first research, individuals treated with citalopram had a 45.5% improvement in HDRS score from baseline to endpoint, compared to 16% improvement in those treated with placebo. Although the citalopram group had a greater drop-out rate, the observed adverse effects were well tolerated and transitory [29]. Rampello et al. found that citalopram was more effective in anxious depressed individuals, whereas reboxetine was more beneficial in retarded depressive patients in the second experiment. During the research, no serious adverse effects were observed. The drop-out rate was also comparable between groups (three for each) [30].

Four trials looked examined the efficacy of fluoxetine, another SSRI, in both the early and late stages of stroke recovery [27,31-33]. The findings of this research are inconclusive. While Fruehwald and coworkers only noticed such a positive effect in the follow-up, Wiart and colleagues observed a positive effect on mood even in the early stages, with a mean improvement in Montgomery-Asberg Depression Rating Scale (MADRS) score of 58% for the fluoxetine group vs. 31% for the placebo group. Robinson and coworkers discovered that fluoxetine was less beneficial than nortriptyline, while Choi-Kwon and colleagues found that it was only helpful for emotional incontinence and wrath proneness. The drop-out rates were also variable: Fruehwald observed no drop-outs during therapy, Wiart observed only two (13.3%) in patients receiving fluoxetine, Choi-Kwon recorded 19.7% in the fluoxetine group and 15.8% in the placebo group, and Choi-Kwon recorded 15.8% in the placebo group [31-33].

Sertraline, another SSRI antidepressant, did not affect major depressive episodes or mild depressive disorder when compared to placebo [34]. In terms of a significant episode of depression or minor depressive illness, as well as short- and long-term antidepressant effects and cognitive outcomes, there were no differences between the therapy. But only after 26 weeks, when the compound was monitored, did it show a noticeable improvement in Quality of Life (QoL). There were no major negative effects reported [34].

Lauritzen and colleagues examined the effectiveness of two tricyclic antidepressants (TCAs), desipramine, and imipramine when both were coupled with mianserin in a trial. The medication doses were changeable, and therapy was directed by side effects. Desipramine therapy was less effective than imipramine therapy in reducing depressive symptoms as assessed by the Melancholia Scale but not by the HDRS. Nevertheless, a sizable portion of the sample (35%) was lost during follow-up, particularly in the desipramine group [35].

In a controlled investigation, Reding and colleagues assessed the response of depressive symptoms to trazodone against placebo. They found that, as measured by the Barthel index, trazodone-treated patients exhibited a better level of autonomy in their capacity to carry out activities of daily life than placebo-treated patients. However, both groups of patients had a substantial drop-out rate due to adverse effects. Particularly, 12 patients left the study: six from the trazodone group (four due to sleepiness, one due to eye discomfort, and one due to refusal), and six from the placebo group (four due to sleepiness, one nausea, one dizziness). Furthermore, due to the study's design, it was unable to compare the two groups' improvements in depression ratings [36]. The details of all these studies are presented in Table 4.

**Table 4:** Studies on the treatment of Post-Stroke Depression that are double-blind and controlled. BDI – Beck Depression Inventory; HDRS – Hamilton Depression Rating Scale; MADRS – Montgomery Asberg Depression Rating scale; STAS – Spielberger Trait Anger Scale; ZSRDS – Zung Self-Rating Depression Scale

Authors	Number of Patients	Treatment Studied	Design	Time from stroke	Trial length	Outcome measures	Results
Lipsey et al.1984 [26]	34	Nortriptyline vs Placebo	Double-blind	<18 months	4-6 weeks	HDRS, ZSRDS	Nortriptyline was shown to be more effective than a placebo in treating depression.
Robinson et al.2000 [27]	56	Nortriptyline vs Fluoxetine vs Placebo	Double-blind	4-16 weeks	12 weeks	HDRS	The response rate to nortriptyline was much higher than that of fluoxetine or placebo.
Anderson et al.1994 [29]	66	Citalopram vs Placebo	Double-blind	2-52 weeks	16 weeks	HDRS	Citalopram was shown to be more effective than a placebo in treating depression.
Rampello et al.2004 [30]	74	Citalopram vs reboxetine	Double-blind	<12 months	16 weeks	HDRS, BDI	Citalopram is more effective in anxious depressive individuals, whereas reboxetine is more beneficial in depressed patients who are retarded.
Wiart et al.2000 [31]	31	Fluoxetine vs placebo	Double-blind	<3 months	6 weeks	MADRS	Fluoxetine was shown to be more effective than a placebo in treating depression.
Fruehwald et al.2003 [32]	54	Fluoxetine vs Placebo	Double-blind	<2 weeks	3 months	HDRS, BDI	At an 18-month follow-up, fluoxetine was found to be more beneficial than a placebo.
Choi-Kwon et al.2006 [33]	152	Fluoxetine vs placebo	Double-blind	14 months	3 months	BDI, Clinical, STAS	Only in the treatment of emotional incontinence and rage proneness is fluoxetine more effective.
Murray et al.2005 [34]	123	Sertraline vs Placebo	Double-blind	3 days – 1 year	26 weeks	MADRS, EDS	Only in terms of emotional anguish, emotionalism, and quality of life is Sertraline better.
Lauritzen et al.1994 [35]	20	Imipramine+mianserin vs desipramine+mianserin	Double-blind	<3 months	6 weeks	HDRS	Imipramine with menazine is more effective than desipramine plus menazine.
Reding et al.1986 [36]	27	Trazodone vs placebo	Double-blind	6 weeks	4-5 weeks	ZSRDS	Trazodone trend toward the better functional status

Finally, the American Heart Association advises using antidepressants for poststroke depression, which should be continued for at least six months following recovery [37].

Prevention

To enhance long-term patient prognosis, secondary stroke prevention focuses on preventing stroke recurrence [38].

**Figure 2:**
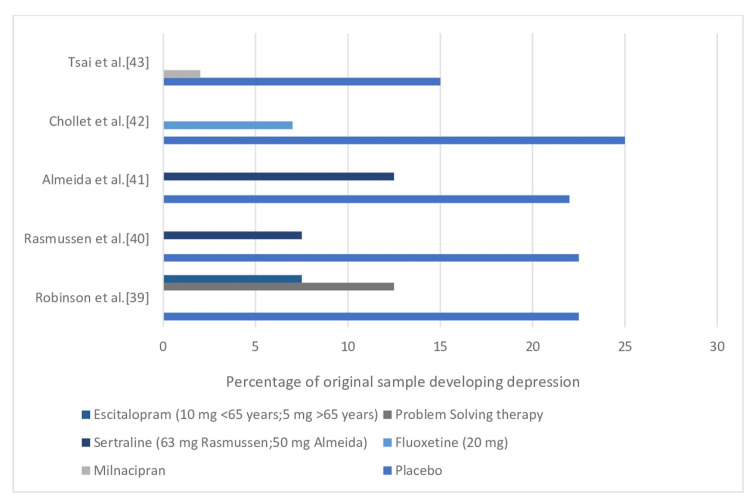
Trials of Preventative Treatments for Poststroke Depression in Randomized Controlled Trials.

The demonstration of preventative therapy is perhaps the most significant advancement in the treatment of poststroke depression (Figure 2). Robinson et al. [39], published in 2008, conducted the first statistically significant randomized controlled trial of prevention of PSD, in which 58 nondepressed acute stroke patients were treated with escitalopram (5 mg/day for patients over 65; 10 mg/day for patients 65 and under) had an incidence of PSD of 8.5% over a year, compared to 11.9% for 59 patients receiving problem-solving therapy and 22.4% for 59 patients receiving placebo. Controlling for age, gender, stroke severity, and disability severity, the risks of developing depression in placebo patients were more than four times higher than in escitalopram patients (adjusted hazard ratio=4.5; 95% CI=2.4-8.2, p<0.001) [2]. Robinson et al. [39], Rasmussen et al. [40], Almeida et al. [41], Chollet et al. [42], and Tsai et al. [43] studies had adequate power to establish statistical significance, even though the percentage of patients experiencing depression with placebo or pharmaceutical therapy was relatively comparable.

The results of eight randomized controlled trials evaluating the efficacy of preventative therapies among 776 originally non-depressed stroke patients were presented in the most current meta-analysis of prevention studies [44]. According to pooled studies, the risk of acquiring poststroke depression was lower in participants undergoing active pharmacologic therapy, especially after a year, and when an SSRI was used [2].

Limitations

The papers considered in this study were all published in English and Spanish between 2012 and 2022 and were found in five databases. Grey literature and other databases were excluded from the study. The study's observational approach limits it to showing connections between variables but not causal or temporal relationships. There is no information on clinical diagnosis of depression using structured interviews or the incidence of departures from standard Center for Epidemiological Studies standards since this study used existing data sets. It's unknown how Depression Scaale is administered. As a result, future studies on people who have had a stroke will benefit from interview evaluations of depression and stroke risks that are tailored to early identification and treatment.

## Conclusions

Our findings indicate the necessity for routine assessments and active screening for depressive symptoms in stroke survivors, as well as the early initiation of therapy to reduce morbidity. Due to the overlapping symptoms of stroke and depression, PSD is frequently misdiagnosed early in the disease's course, resulting in the patient's functional and social status being harmed. As a result, using the numerous diagnostic methods indicated above, poststroke depression should be identified early. There is evidence that antidepressant medications can help treat depression, but they cannot achieve full clinical remission or prevent depressive disorders from developing. Identifying further features of the poststroke depression process, we believe, is the most pressing need for future study since it might lead to a more precise treatment strategy. Because the advantages of antidepression treatment might be significant, not only in terms of mood but also in terms of functional recovery, further study in this area of stroke medicine is urgently needed.
